# A MISO UCA Beamforming Dimmable LED System for Indoor Positioning

**DOI:** 10.3390/s140202362

**Published:** 2014-01-29

**Authors:** Attaphongse Taparugssanagorn, Siwaruk Siwamogsatham, Carlos Pomalaza-Ráez

**Affiliations:** 1 Wireless Information Security and Eco-Electronics Research Unit/National Electronics and Computer Technology Center, Bangkok 12120, Thailand; E-Mail: siwaruk.siwamogsatham@nectec.or.th; 2 Radio Frequency (RF) Communications, Purdue University, Fort Wayne, IN 46805, USA; E-Mail: cpomalaz@purdue.edu

**Keywords:** indoor location awareness, optical wireless communications, adaptive beamforming

## Abstract

The use of a multiple input single output (MISO) transmit beamforming system using dimmable light emitting arrays (LEAs) in the form of a uniform circular array (UCA) of transmitters is proposed in this paper. With this technique, visible light communications between a transmitter and a receiver (LED reader) can be achieved with excellent performance and the receiver's position can be estimated. A hexagonal lattice alignment of LED transmitters is deployed to reduce the coverage holes and the areas of overlapping radiation. As a result, the accuracy of the position estimation is better than when using a typical rectangular grid alignment. The dimming control is done with pulse width modulation (PWM) to obtain an optimal closed loop beamforming and minimum energy consumption with acceptable lighting.

## 1. Introduction

In recent years, light emitting diodes (LEDs) have attracted great interest for landscape architecture and illumination applications because of their characteristics such as long life expectancy, high tolerance to humidity, low power consumption, light quality, color rendering, and environmental friendliness [1]. Although LEDs have several advantages, light control is still very important to improve the quality of lighting. Light control is the ability to regulate the level of lighting in a given space for specific tasks or situations. Controlling light properly not only helps to save energy, but also enhances lighting satisfaction, which is highly correlated to mood and productivity. Lighting accounts for 25%–30% of energy use in a building's electrical system [2]. In an average home or office, most light controls are just simple ON/OFF switches. This means that whether it's the middle of the day or it's nighttime, the fixtures are putting out the exact same amount of light. Unlike just ON/OFF, through dimming, users can control the quantity of light to fit specific tasks, moods, or situations [2].

Two major approaches to make LEDs dimmable are pulse width modulation (PWM) and analog dimming. Both methods control the time-averaged current through the LED or LED string, but they have differences which become evident when examining their advantages and disadvantages. PWM dimming greatly reduces color changes in the LED with varying brightness levels, because the LED essentially runs at a constant current when it is on and at no current when it is off. However, this advantage comes at the expense of additional logic to create the PWM waveforms [3]. On the contrary, analog dimming can use a simpler circuit, but the variable current supplied to the LED means that the regulator supplying the current to the LED must consume any power not supplied to the LED. This additional power leads to undesirable heat-generating energy waste. In addition, analog dimming may be inappropriate for applications that require a constant color temperature [3].

Recently, location awareness, in particular, for indoor environments, has drawn interest from researchers in many innovative applications of wireless systems, for instance, positioning or tracking people or objects inside buildings. Several existing indoor localization approaches use radio frequency (RF) signals [4–7]. Practical RF systems usually have the situation of multipath fading and non-line-of-sight (NLOS) conditions in indoor scenarios, therefore it is difficult to measure precise distances using the received signal strength, and the estimated position error can be on the order of meters. It is possible to utilize Ultra Wide Band (UWB) technology to measure precise ranges based on time-difference-of-arrival (TDOA), but this technology requires complicated hardware [8]. Moreover, the use of RF-based positioning systems is restricted in places such as hospitals, kindergartens, airplanes, and areas with RF-sensitive equipment. To overcome these problems the use of visible light, an emerging technology in which LEDs transport information wirelessly, has been proposed [1]. LED light can be used for identification and localization systems, known as LED-ID localization. An LED-ID system includes at least two components, an LED-ID reader and an LED-ID transmitter (tag). The LED-ID reader receives the required information from the LED-ID transmitters. LED-ID systems are very suitable in RF interference-sensitive areas and therefore they are attractive since both illumination and localization purposes can be simultaneously provided. In addition, the use of LEDs is safer to human eyes, due to the intense visible light triggering the blinking reflex, and then preventing a prolonged exposure. This allows for an increase in the transmission power, high illumination levels, better coverage and link robustness, even in NLOS scenarios.

A multiple input single output (MISO) beamforming system has been considered in several standards for next generation wireless communications systems. With codebook-based feedback, these systems can potentially achieve the same diversity order and a larger coding gain when compared to non-feedback systems like space-time codes [9–12]. An optical beamforming based on the appropriate phase shifts has been proposed to create bright spots in a service area [13]. With a proper design of radiation patterns, the system can enhance the bit error rate (BER) performance and enlarge the coverage area.

Besides the illumination purposes of dimmable LEDs, in this paper, the advantage of dimming is studied for positioning purposes. The LEDs start from a certain brightness level and increase their brightness level until the LED reader can detect the nearest light emitting array (LEA) using a MISO beamforming technique that is discussed in details later. Note that the positioning service works even if there is no dimming control or brightness change. When the dimming control is applied, a reliable (more accurate) positioning estimation can be achieved by the optimal closed loop beamforming. Consequently, the light beam from the LEA is supposed to be the minimal energy detectable beam. While positioning and/or communications can be achieved using dimmable LEDs, the brightness level can also satisfy the users [14].

The organization of the paper is as follows: Section 2 presents the model for LED-ID positioning system in a simple square room. However, all ideas and techniques can easily extend to more complex indoor environments, *i.e.*, non-rectangular shaped rooms, corridors, *etc*. Both the typical rectangular grid shape and a hexagonal lattice alignment are deployed. In Section 3, a MISO system with *M* LEDs (transmitters) and one photo-detector (receiver), performing a closed-loop transmit beamforming technique, is described. Section 4 describes the results of simulation studies. In Section 5, the issue of actual implementations is discussed. Conclusion remarks and future plans are included in Section 6.

## 2. Signal and System Models

**Figure 1.**
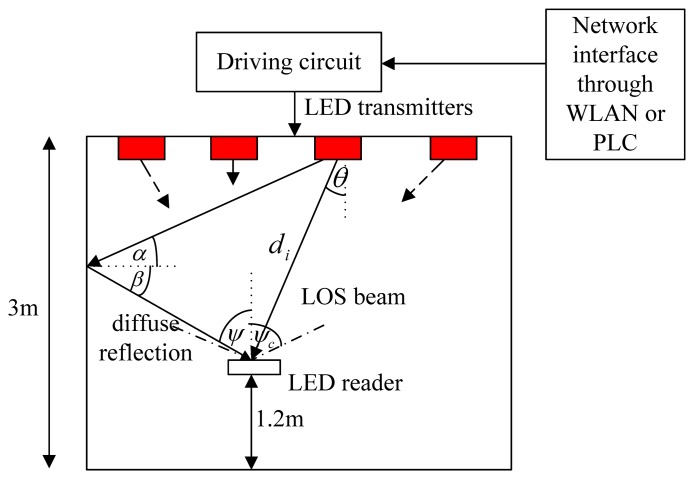
Propagation model of optical wireless channels.

**Figure 2.**
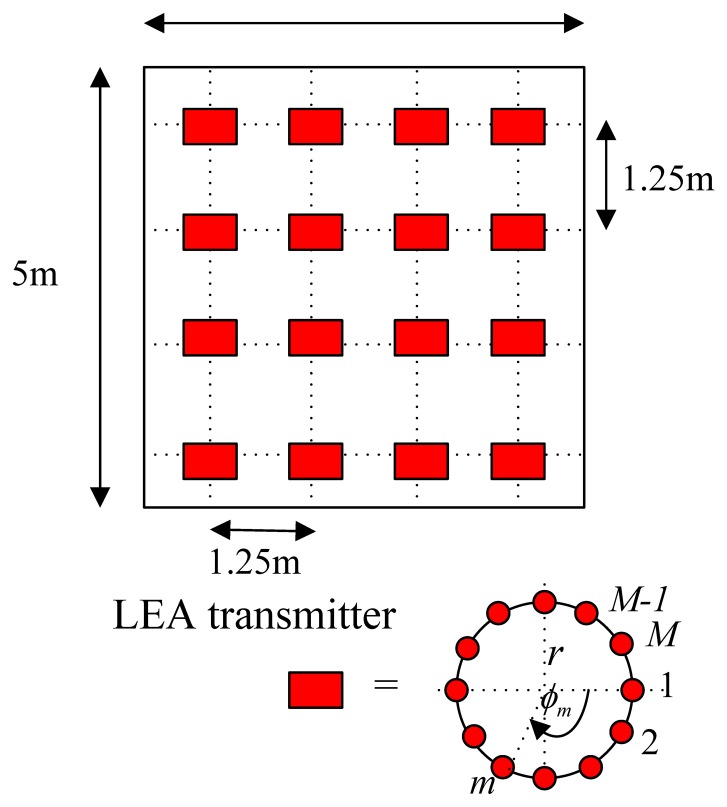
LEAs' placement on the ceiling in a typical rectangular grid.

**Figure 3.**
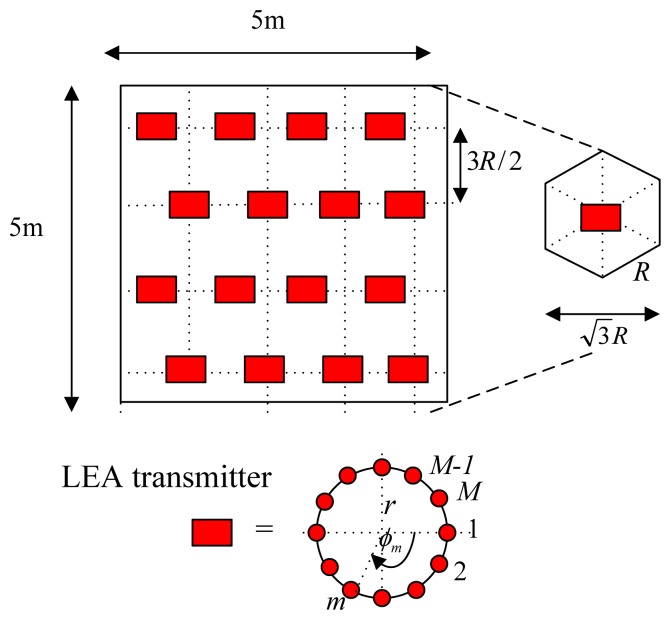
LEAs' placement on the ceiling in a hexagonal lattice alignment.

The system is deployed in a room with dimensions 5 × 5 × 5 m^3^ (width, length and height). All the walls are white, have a constant reflection coefficient throughout and are modeled as a first order Lambertian source. There are sixteen dimmable light emitting arrays (LEAs) as transmitters located on the ceiling. Each LEA is a uniform circular array (UCA) consisting of *M* LED bulbs. A UCA geometry is used due to its symmetrical configuration which enables the phased array antenna to scan azimuthally with minimal changes in its beam width and sidelobe levels. The array factor (*AF*) at a far-field of (*θ*_0_,*ϕ*_0_) is given by:

(1) $$AF\left(\theta ,\varphi_{m}\right)=\overset{M}{\underset{m=1}{\text{∑}}}a_{m}\exp\left(\text{jlr}\sin\theta \cos\left(\varphi_{0}-\varphi_{m}\right)\right)$$

where *a_m_* and *ϕ_m_* are the amplitude excitation and the azimuth angle of the *m*^th^ element, and *l* = 2*π/λ*. A far-field assumes that the target is farther from the source than this nominal separation, and the source can be modeled or measured as an emitting point. The “5 times” rule of thumb is valid for a source with circular shape and Lambertian emission. This rule states that for a distance of five times the source diameter, the error from using the inverse-square law is 1%. Therefore, the far-field condition can be assumed for this application. The reader can be assumed to be located in the far field of LEDs [15]. The zenith angle is represented by *θ* as shown in Figure 1. The UCA is designed optimally to produce zero-order Bessel-like or Bessel-Gauss patterns. A Bessel beam is also named as nondiffracting beam [16,17]. In addition to the typical rectangular grid shape, the LEAs' placement is also deployed in an hexagonal lattice alignment as proposed in [18], both are illustrated in Figures 2 and 3. The LED reader, e.g., photodiode or image sensor, which can be plugged into a smartphone, is held by a tracking person at the height of 1.20 m, *i.e.*, 1.80 m below the ceiling. Each dimmable LED bulb can be not only remotely controlled with an app on the smartphone, but it can also be linked to keypads, motion sensors or door sensors, to ensure that the lights are used when people are in the room.

For simplicity, the discrete form for our system is used. At time instant *k*, the system can be modeled as a linear time-invariant system having the impulse response *h*[*k*] together with additive white Gaussian noise (AWGN) *W*[*k*] representing the noise at the optical receiver, e.g., for avalanche photo diode receiver *W*[*k*] including both shot noise and thermal noise with zero mean and variance $\sigma_{W}^{2}$ as:

(2) $$P_{R}[k]=F\sum_{i=1}^{M}\left(P_{\text{T}i}[k]w_{i}*h_{i}[k]\right)+W[k]$$

where *P*_T_*_i_*[*k*] and *P*_R_[*k*] are the instantaneous transmitted optical power on the *i*^th^ LED in an LEA and the instantaneous optical power or the intensity on the photodetector, respectively. Before each LED in an LEA emits the optical signal, the RF signal is multiplied with a weight vector w = [*w_0_*, *w_1_*, …, *w_m_*] through a dimming control using pulse width modulation (PWM) as explained in the next subsection. The photodetector responsivity or optical/electrical conversion efficiency is denoted by *F*. The symbol * denotes a convolution integral.

### 2.1. Signal Modulation for Dimming Control

**Figure 4.**
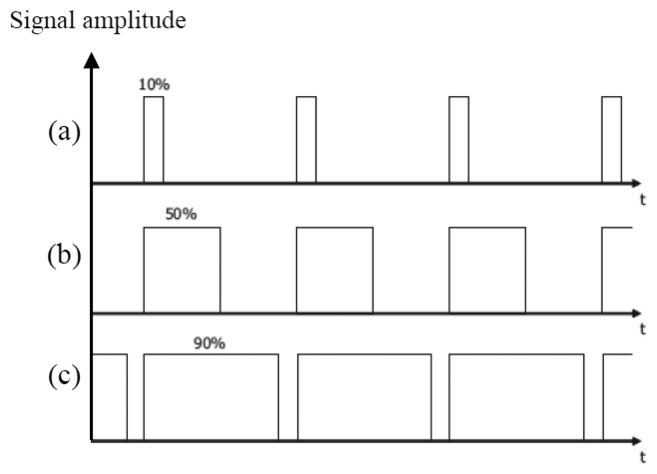
Pulse Width Modulation.

*P_T_*(*t*) is a pulse width modulated signal. PWM is a way to digitally encode analog signal levels [18]. PWM is used in motor drive circuits, dimmer control, power control, *etc*. The LED current is modulated by PWM signal to control its brightness changing the “ON” duration of the whole period, while the LED current is kept constant. During the “OFF” period, there is no signal transmitted. The duty cycle *D* of PWM signal is varied according to how often is the “ON” and the “OFF” periods. When the duty cycle is 100%, all the LED light is transmitted and the light is with its highest brightness. When the duty cycle is reduced, the LED light is blocked in the “OFF” duration. Therefore, the light is dimmed when the whole period of a PWM signal is taking into account. Figure 4a–c shows examples of three different PWM signals, *i.e.*, with the duty cycle 10%, 50%, and 90%, respectively. This “ON”-“OFF” flashing condition does not affect what is seen as the human eyes fills in the gaps between the “ON” and “OFF” light pulses, provide that the pulse frequency is high enough (at least 200 Hz) to make it appear as a continuous light output [19]. When using a PWM signal, the total number of transmitted bits is reduced, which deteriorates the overall system performance. In order to maintain the communication quality in terms of the number of transmitted bits under dimming control scheme, the data rate of the modulating signal should be adjusted when the duty cycle of PWM dimming control signal is changed. Although the BER performance (as discussed later) during the “ON” duration of the PWM dimming control signal is not changed, taking into account the whole PWM period the number of transmitted bits is reduced. In order to keep the number of transmitted bits unchanged when the LED light is dimmed, the data rate should be increased as:

(3) $$\begin{array}{l} R_{\text{d}}TD=R_{\text{o}}T,\\ R_{\text{d}}=\frac{R_{\text{o}}}{D} \end{array}$$

where *R*_o_ is the original data rate, *R*_d_ is the data rate under dimming control, and *T* is the signal period.

The main reason for the use of the PWM technique is that it is simple to implement, requiring only that the backlight can be switched on and off rapidly, and also it gives a large range of possible luminance values. This technique also does not result in a loss of efficiency. Under dimmed conditions, the LEDs are still operating at the same voltage and current as during full light output. In addition, PWM gives a more continuous level of light than other methods [19].

### 2.2. Wireless Channel Model for LED-ID System

It is assumed that an LED has a Lambertian radiant intensity:

(4) $$R_{0}(\theta )=\left[\left(m+1\right)/2\pi \right]cos^{m}(\theta )$$

where *m* is the order of Lambertian emission, and is related to the transmitter semi-angle (at half power) *θ*_1/2_, as *m* = −ln 2/ln(cos *θ*_1/2_) [20]. *P*_LED_ is the power emitted by an LED. *θ* and *ψ*, shown in Figure 1, are the irradiance (or zenith) and incidence angles, respectively. After modulation, the optical signal from an LED is given by:

(5) $$P_{\text{LED}}[k]=P_{\text{T}}(1+M_{\text{I}}f[k])$$

where *P*_T_ is the LED output power without modulation, *M*_I_ is the modulation index, and *f*[*k*] is the normalized modulating signal. The modulation index is defined as the ratio of the LED's maximum current variation caused by the modulating signal to the LED bias current [21]. Because the walls are assumed to act as large reflecting surfaces, the light beams propagate from a LED transmitter to the LED reader via two main paths, *i.e.*, line of sight (LoS) and diffuse channels. Since the rate of change of the channel impulse response is very slow compared to the frequency of the optical signal, it is usually sufficient to use a static parameter (channel DC gain) to represent the channel [14]. The DC gain can be estimated accurately by considering only the LoS path [21]. The channel transfer function of the DC gain [the Fourier transform of the LoS part of *h*[*k*] in (2)] at *f* = 0 is expressed as:

(6) $$H_{\text{LOS}}(0)=\{\begin{array}{cc} \frac{A}{d^{2}}R_{0}(\theta )\cos\psi , & 0\leq \psi \leq \psi_{c}\\ 0, & \psi >\psi_{c} \end{array}$$

where *A* is the detector area, *d* is the distance between the LED transmitter and the LED reader, and *ψ_c_* is the field-of-view (FOV) of the LED reader.

For a diffuse channel, the impulse response of the diffuse signal *h*_diff_[*k*] shown in Figure 3 in [21] consists of some initial peaks due to the first reflections in the room and then a smooth exponential decay caused by the higher order reflections. This was modeled by an integrating sphere model [22,23]. When both the LoS path and the diffuse channel are considered together, *h*[*k*] is modeled as a Rician *K*-factor distributed random variable, which is widely used in radio communications. The average received optical power at the reader is given by *P*_R_ = *P*_T_*H*_LOS_(0). After the photodetection in the reader, the DC component of the detected signal is removed, and the output signal is given by:

(7) $$P_{\text{F}}[k]=FP_{\text{R}}M_{\text{I}}f[k]+W_{\text{F}}[k]$$

where *W*_F_[*k*] is the noise after the filter and is assumed to be AWGN with zero mean and variance $\sigma_{W_{F}}^{2}$. Thus, the output SNR *γ*_o_ is given by [23]:

(8) $$\gamma_{\text{o}}=\frac{{\left(FH_{\text{LOS}}(0)P_{\text{T}}M_{\text{I}}\right)}^{2}\langle f{\left[k\right]}^{2}\rangle }{\sigma_{W_{\text{F}}}^{2}}$$

where 〈·〉 is the time average operator.

The BER performance of the signal is expressed as:

(9) $$\begin{array}{ll} \text{BER} & =Q\left(\sqrt{2\gamma_{\text{o}}}\right)\\ & =Q\left(\frac{\sqrt{2}FH_{\text{LOS}}(0)P_{\text{T}}M_{\text{I}}}{\sigma_{W_{\text{F}}}}\right) \end{array}$$

## 3. Transmit MISO Beamforming

A MISO system with *M* LEDs (transmitters) and one photodetector (receiver) performing a closed-loop transmit beamforming technique is considered. As previously described, dimming can be used to control the quantity of light to fit specific tasks, moods, or situations. Likewise, dimming can be used for localization tasks by incorporating closed-loop beamforming techniques. The weight vector **w** is a complex vector corresponding to the inverse of the phase of the channel to ensure that the signals add constructively at the receiver, *i.e.*, *w_i_* = exp(−*jϕ_i_*) with *h_i_*[*k*] = |*h_i_*|exp(*jϕ_i_*). *h_i_*[*k*] for each LED can be written together in the form of vector as h[*k*]. The weight vector is a predetermined codebook, known to both the transmitter and receiver prior to any communication. Furthermore, the codebook is considered to be fixed throughout the communication time. Since the channel is time-varying and unknown a priori, the receiver has to estimate the channel based on training signals. The channel estimation is assumed to be perfect. The channel estimate at the receiver at time *k-D*, where *D* is the feedback delay, is quantized into one of the codewords in the codebook. In theory the quantization is performed via an exhaustive search over the codewords in the Grassmannian codebook [11] as:

(10) $$b=\underset{1\leq i\leq M}{\arg\max}{\left|\hat{\text{h}}\left[k-D\right]{\text{w}}^{H}\right|}^{2}$$

where the index *b* is fedback to the transmitter and the transmitter chooses vector **w***_b_* for beamforming (an error-free feedback channel is assumed). In an actual implementation, the Grassmannian codebook can be obtained from a lookup table, *i.e.*, an exhaustive search is not really required. *N* = 2*^B^* is the number of the beamforming vectors in the codebook with *B* bits of feedback Hence, with a feedback delay of *D*, the beamforming weight vector used at time *k* is **w**[*k*] = **w***_b_*. A weight vector is matched to the corresponding PWM signals for dimming control. The output SNR of the transmit MISO beamforming system, which corresponds to the output SNR *γ*_o_ in Equation (8) is given by:

(11) $$\gamma_{\text{o}}=\gamma_{i}{\left|\text{h}{\text{w}}_{i}\right|}^{2}$$

where *γ_i_* denotes the transmit SNR given as $\gamma_{i}=\frac{P_{{\text{T}}_{i}}}{\sigma_{w}^{2}}$ with the total transmit power *P*_T_*_i_*. In terms of positioning, the direction of departure (DoD), *i.e.*, *θ*, *ϕ_i_*, and the distance between a transmitter and the receiver *d_i_* in Figure 1 are used for calculating the position of the receiver.

## 4. Simulation and Analysis

The performance of the proposed localization system is evaluated using Monte-Carlo simulations. The results of positioning for both type of alignments, *i.e.*, a rectangular grid and a hexagonal lattice, are compared. All the parameters are given in Table 1. The LED reader or the receiver is assumed to be at a fixed height of 1.20 m, *i.e.*, 1.80 m below the ceiling for the entire simulations.

**Figure 5.**
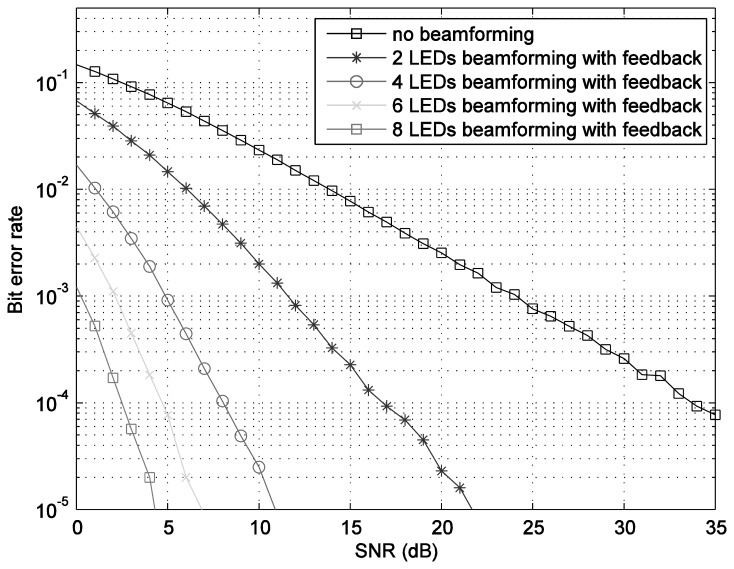
Bit error rate (BER) performance *versus* SNR for MISO transmit beamforming.

The optimum indexed codebooks are configured by using the given beamforming vectors. The bit error rate BER performances, shown in Figure 5, are calculated by using Monte-Carlo simulation with the assumption of perfect feedback, *i.e.*, error-free feedback. As it can be observed, the transmit beamforming technique significantly enhances the performance compared to the one without beamforming. The number of LEDs in each LEA proportionally increases the performance.

**Figure 6.**
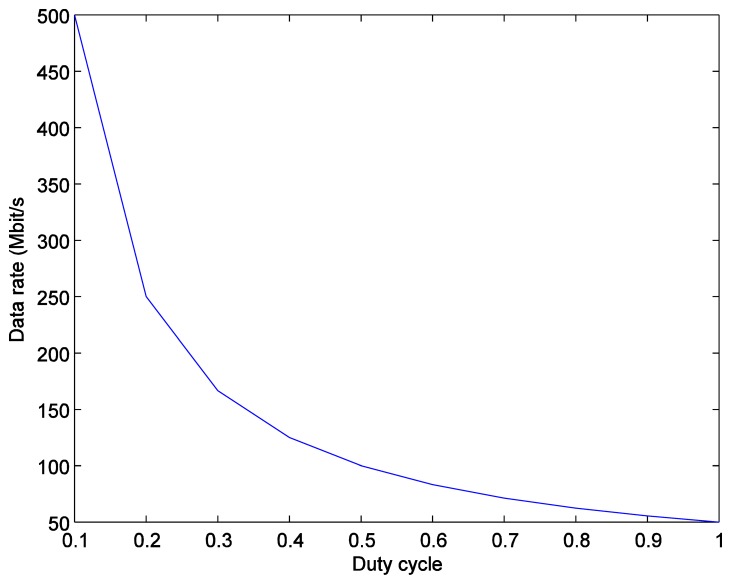
Adaptive data rates *versus* the duty cycles.

As expressed in Equation (3), under a dimming control scheme the adaptive data rate is inversely-proportional to the duty cycle of the PWM signal in order to make the number of transmitted bits constant. Figure 6 then shows that the data rate *R*_d_ becomes higher than the original data rate *R*_o_ as the duty cycle becomes smaller.

**Figure 7.**
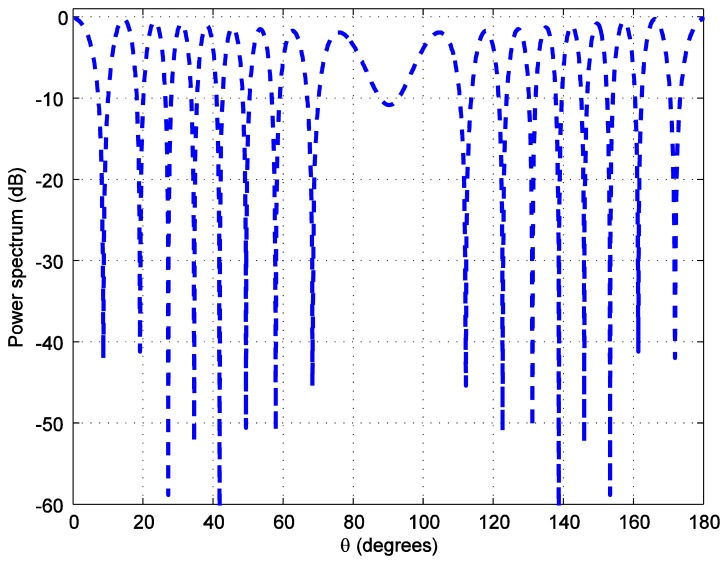
Transmit power spectrum *versus* zenith angle *θ* for *M* = 2 bulbs.

**Figure 8.**
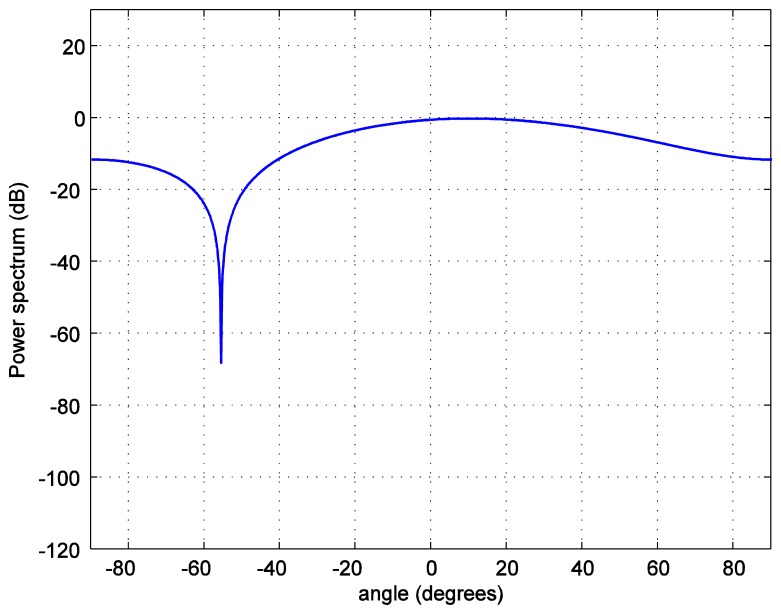
Transmit power spectrum *versus* azimuth angle *ϕ* for *M* = 2 bulbs.

**Figure 9.**
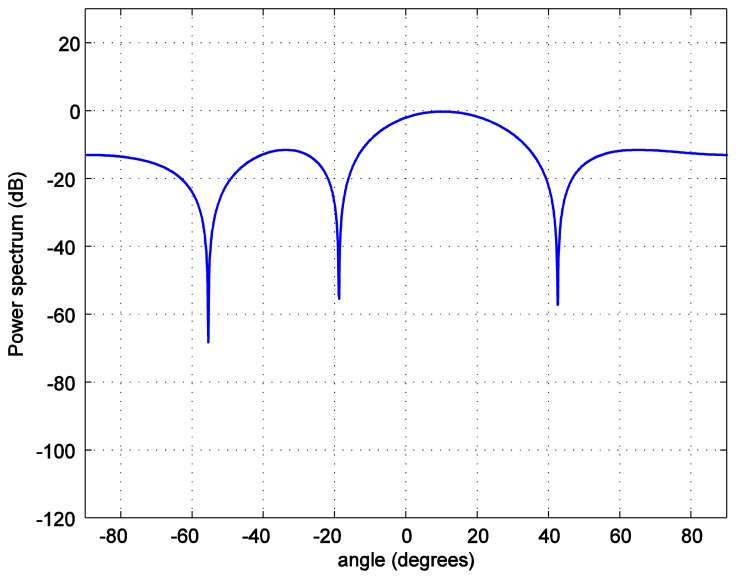
Transmit power spectrum *versus* azimuth angle *ϕ* for *M* = 4 bulbs.

**Figure 10.**
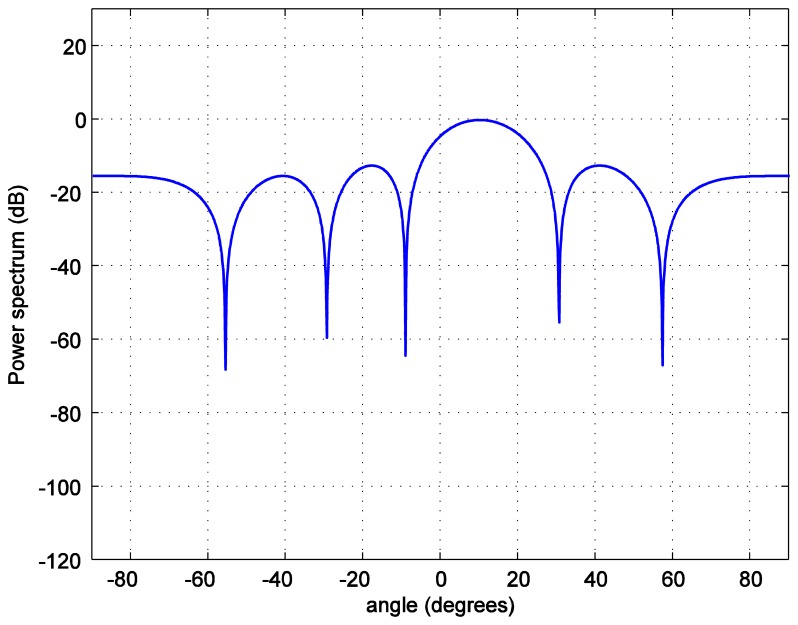
Transmit power spectrum *versus* azimuth angle *ϕ* for *M* = 6 bulbs.

**Figure 11.**
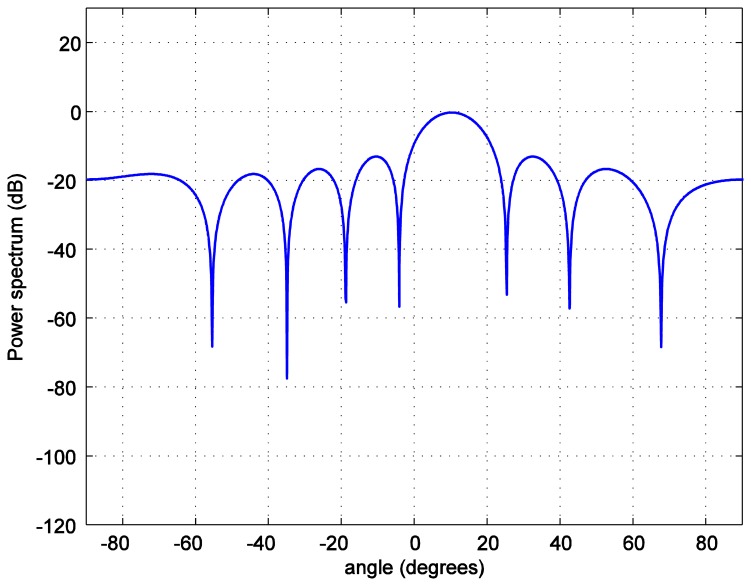
Transmit power spectrum *versus* azimuth angle *ϕ* for *M* = 8 bulbs.

For the positioning calculations, the angle *ϕ* is set to 10 degrees. One hundred thousand positions of the LED reader are generated starting from the position at (0.0158, 0.0158, 1.2) and increasing 0.0158 m in each step in both, *x* and *y*, axes covering the whole room. To investigate the accuracy of the position estimation, the array radiation pattern or the power spectrum *PS* is calculated as:

(12) $$PS=AF\left(\theta ,\varphi_{m}\right)R\left(\theta ,\varphi_{m}\right)$$

where *R*(*θ*,*ϕ_m_*) is the radiation pattern of each antenna. The power spectrum *versus* the zenith angle for the number of LEDs in each LEA, *M* = 2 bulbs is plotted in Figure 7. The number of LEDs in each LEA varies as 4, 6, and 8. As it can be seen in Figures 8, 9, 10 and 11, the larger the number of LEDs used, the clearer the main lobe of the angular spectrum becomes. This result can also be express in that the number of LEDs in each LEA proportionally increases the BER performance as shown in Figure 5. Therefore, the position estimation performance increases as the number of LEDs increases according to the relationships given in Equations (8–11). However, a practical number and sufficiently good performance is desirable, e.g., eight bulbs. The reader, which is assumed to have a received signal strength indicator (RSSI) coupled directly into the microcontroller where the decision is made over which input has the strongest signal, can indicate which LEA gives the strongest signal for each position of the reader [24].

In addition, the position estimation performances of both types of alignments are compared using both the absolute mean error (average of the absolute error) and the root mean square error, RMSE (square root of the mean squared error). The results in Table 2 show that with the same number of LEDs (e.g., eight bulbs) a hexagonal lattice alignment provides a more uniform illumination rendering than the one with a square or a triangular grid. The explanation for these results is that the coverage holes and overlapping radiation areas are reduced when a hexagonal lattice alignment is used. This means that a hexagonal lattice alignment can be a better choice for all illumination, positioning, and communications applications.

## 5. Discussion for Actual Implementations

Currently there are two following types of white LEDs on the market: 1-chip LEDs (blue LED chip with yellow phosphor) and 3-chip LEDs (red, green, and blue chips) [25]. The former one is dominant due to its simple mass production. Nevertheless, a major challenge in utilizing these 1-chip white LEDs to achieve high data rate communication is its limited modulation bandwidth, usually in the range of several MHz without equalization. A pre-equalization of a white LED to further increase the modulation bandwidth is needed for high data rate communication systems [26].

A typical indoor visible light communication receiver usually consists of a concentrator, an optical filter, a photodetector, a pre-amplifier, a post-equalizer, and an electrical filter. For NLOS channel such as a diffuse channel, using non-imaging concentrator and corresponding optical filter could effectively enlarge the active receiving area and broaden the FOV to increase the received optical power. However, for the directed LOS channel, the FOV should be designed to be small to reduce the received ambient light noise power. The ambient light noise is usually diffused inside the whole room as background light [21]. Typically, there are two types of photodetectors adopted in an indoor LOS visible light communication system, *i.e.*, the photodiode and the image sensor [21]. The photodiode has been widely used when the received optical power is relatively high. The advantages of the photodiode are low price and possible high reception bandwidth. On the other hand, the image sensor is able to provide receiver spatial diversity to enhance detection performance and additional source location information for location-aware services [21]. For application scenarios where multiple LED arrays in a room send different signals to multiple users, using a large FOV photodiode detector may lead to large interference that degrades received SNR. In this case an image sensor would better used as a photodetector [21].

The Media Access Control (MAC) specifications include addressing and channel access control mechanisms to allow for several terminals or readers. The LEDs send their IDs frequently as a type of a beacon signal at different wavelengths. A beacon signal is used to indicate the start of frame and define the number of slots to be allocated for contention, uplink and downlink. There is also non-beacon mode used only for the peer-to-peer (P2P) application. These beacon signals are usually sent at the lowest clock rate C_1_ (200 kHz), which is mandatory at both the transmitter and receiver for all devices [27]. For every clock rate, there is an associated set of data rates at the physical layer. This data rate is dependent on the modulation, line coding and Forward Error Correction (FEC) used at the physical layer for a given clock rate. In [27], there is a data rate table rate {C*_i_*_,_*_j_*} associated with each clock rate C*_i_* where *j* is the data rate index associated with clock rate C*_i_*. The data rate table is a common standard adopted by all VLC devices [27].

The reader is assumed to have a received signal strength indicator (RSSI) unit coupled directly into the microcontroller where the decision is made over which input has the strongest signal. Therefore, the reader can indicate which LEA gives the strongest signal for each position of the reader. A measure of the received signal strength indication (RSSI) is coupled directly into the microcontroller (μC) where the decision is made over which input has the strongest signal. This information is translated to a binary address associated with the data inputs of the analogue multiplexer [24]. The multiplexer output is sent directly to the clock and data recovery module [24]. In the case where the signal on the LOS detector has been blocked, the μC can send a signal to the RF uplink unit requesting that the VLC transmitter increase or decrease the transmission modulation depth [24].

Unlike in a simulation, the required transmit channel knowledge for the weight vector adaptation is not perfect. A training sequence with one of the following channel estimation methods, *i.e.*, the correlation method, the least square (LS) method or the minimum mean square error (MMSE) method, can be used. The performance of the correlation method is the worst, the LS method is suitable for high SNR, and the MMSE method obtains the best performance with the highest complexity.

Regarding the feedback of the beamforming, a feedback can be realized in the same way as the brightness control, *i.e.*, the feedback signal is sent by the μC through the RF uplink for adjusting the beamforming vectors in our system [28]. The feedback bits sent from the receiver to the transmitter for beamforming, when the transmitter does not have *a priori* channel knowledge, a limited feedback method, is employed [29]. When implementing limited feedback beamforming, the beamforming vector is restricted to lie in a finite codebook that is known to both the transmitter and receiver. The receiver uses its channel estimate to choose the vector from the codebook that maximizes the conditional receive signal-to-noise ratio (SNR). The binary index of the chosen vector is then conveyed to the transmitter over a limited rate feedback channel. Grassmannian beamforming can be implemented with a lookup table format. When the channel is slowly varying, it may be possible to reduce the necessary number of bits sent back by using some successive refinement techniques based on channel correlation [29].

## 6. Conclusions and Future Work

In this paper, the use of multiple input single output (MISO) transmit beamforming system using dimmable light emitting arrays (LEAs) in the form of a uniform circular array (UCA) as transmitters was proposed. With this technique, visible light communication between a transmitter and a receiver (LED reader) is achieved with excellent performance and with good estimation of the receiver's positions. A hexagonal lattice alignment of LED transmitters was deployed to reduce the coverage holes and the areas of overlapping radiation. As a result, the accuracy of the position estimation is better than the one with a typical rectangular grid alignment. The dimming control was done using pulse width modulation (PWM) in order to obtain the optimal closed-loop beamforming and minimum energy consumption with good lighting satisfaction.

For future work, various circular array configurations can be considered in which each configuration consists of *M* LED bulbs, for instance, uniform circular centered array, planar uniform circular array, and planar uniform hexagonal array [30]. We can expect better position estimations from these array configurations, since they can provide beam patterns with smaller side lobes [29]. Especially, among these array configuration, a planar uniform hexagonal array can provide the narrowest main beam and the smallest side lobes. This is suitable for adaptive array applications which require high spatial resolution [30]. Multiple LED readers can be used and a physical experiment should be carried out to compare the results to the simulation. Ray-tracing based simulations and actual experiments are future plans to compare with the results from the simulations.

## Figures and Tables

**Table 1.** Simulation parameters.

**Parameters**		**Value**
Each LED half power angle (*ϕ*_1/2_*_i_*)		70 degree
Lambertian radiant intensity of each LED (*R*_0_(*θ_i_*))		80 lm
FOV of the LED reader (*ψ_c_*)		180 degree
Transmitted power per LED (*P*_T_*_i_*)		200 mW
LED reader's detector area (*A*)		0.0001 m^2^
Number of LEDs in each LEA (*M*)		2, 4, 6, 8 bulbs
Codebook length (*N*)		4
Number of simulation		10^6^ bits
Photodetector Responsivity (*F*)	1 A/W	

**Table 2.** Absolute mean error and RMSE.

**Coordinates**	**Absolute Mean Error (m)**	**RMSE (m)**
Rectangular *x*	0.23	0.70
Rectangular *y*	0.29	0.75
Hexagonal y	0.16	0.60
Hexagonal y	0.18	0.67
